# Combining Symbolic Cues with Sensory Input and Prior Experience in an Iterative Bayesian Framework

**DOI:** 10.3389/fnint.2012.00058

**Published:** 2012-08-13

**Authors:** Frederike H. Petzschner, Paul Maier, Stefan Glasauer

**Affiliations:** ^1^Institute for Clinical Neurosciences, Ludwig-Maximilians-University MunichMunich, Germany; ^2^Bernstein Center for Computational Neuroscience MunichMunich, Germany; ^3^Graduate School of Systemic Neurosciences, Ludwig-Maximilians-University-MunichMunich, Germany; ^4^Integrated Center for Research and Treatment of Vertigo, Ludwig-Maximilians-University MunichMunich, Germany

**Keywords:** pre-cueing, path integration, cue-combination, multi-modal, categorization, experience-dependent prior, magnitude reproduction, iterative Bayes

## Abstract

Perception and action are the result of an integration of various sources of information, such as current sensory input, prior experience, or the context in which a stimulus occurs. Often, the interpretation is not trivial hence needs to be learned from the co-occurrence of stimuli. Yet, how do we combine such diverse information to guide our action? Here we use a distance production-reproduction task to investigate the influence of auxiliary, symbolic cues, sensory input, and prior experience on human performance under three different conditions that vary in the information provided. Our results indicate that subjects can (1) learn the mapping of a verbal, symbolic cue onto the stimulus dimension and (2) integrate symbolic information and prior experience into their estimate of displacements. The behavioral results are explained by to two distinct generative models that represent different structural approaches of how a Bayesian observer would combine prior experience, sensory input, and symbolic cue information into a single estimate of displacement. The first model interprets the symbolic cue in the context of categorization, assuming that it reflects information about a distinct underlying stimulus range (categorical model). The second model applies a multi-modal integration approach and treats the symbolic cue as additional sensory input to the system, which is combined with the current sensory measurement and the subjects’ prior experience (cue-combination model). Notably, both models account equally well for the observed behavior despite their different structural assumptions. The present work thus provides evidence that humans can interpret abstract symbolic information and combine it with other types of information such as sensory input and prior experience. The similar explanatory power of the two models further suggest that issues such as categorization and cue-combination could be explained by alternative probabilistic approaches.

## Introduction

Because the demands in natural tasks are highly complex but sensory information is corrupted by noise, humans are versed in exploiting contextual information. To improve efficiency, reduce the amount of computational costs, and allow fast adaption to the outside world, we infer existing dependencies and combine relevant information to guide our perception and action. The sources of information can vary from the simultaneous input coming from different senses (Ernst and Bülthoff, 2004; Angelaki et al., 2009) or distinct input from one sensory modality (Jacobs, 1999; Stone et al., 2009), over short and long-term experience (Adams et al., 2004; Stocker and Simoncelli, 2006; Verstynen and Sabes, 2011), to abstract expectations and contextual cues in the environment (Langer and Bülthoff, 2001).

A possible framework for combining these diverse sources of uncertain information is offered by Bayesian probability theory, which has proven applicable to several of the mentioned issues. It provides a normative, mathematical description of how various sources of information can be merged to obtain a statistically optimal estimate of their cause in the presence of uncertainty. One of the most common applications of the Bayesian approach is multi-modal cue integration, where the provided information about a stimulus results from different sensory modalities, such as vision, audition, or proprioception (Ernst and Banks, 2002; Battaglia et al., 2003; Körding et al., 2007).

Senses, however, are not the only source of information that determines our perception. Contextual and symbolic cues can also contribute as a new source of information. In visual search paradigms, contextual cues are known to influence reaction times (e.g., Müller et al., 2003; Vincent, 2011). The context can also lead to an internal organization of stimuli into distinct categories that influence perception by leading to an increased ability to discriminate between categories at the expense of discriminability within categories. Examples for category effects range from the perception of speech sounds (Liberman et al., 1957) or colors (Davidoff et al., 1999) to facial expressions (Etcoff and Magee, 1992). A Bayesian explanation for category effects in speech perception was offered by Feldman et al. (2009). However, their solution only treats implicit predefined categories, not auxiliary contextual cues providing information about these categories, e.g., pre-cueing.

Another type of contextual information comes from the preceding occurrence of a stimulus in the form of prior experience. Bayesian probability theory has been successfully applied to a broad spectrum of studies exploring the effect of short or long-term experience on our current percept (Adams et al., 2004; Stocker and Simoncelli, 2006; Verstynen and Sabes, 2011). For human estimation of distances and turning angles in a production-reproduction task, we have recently shown that the effect of prior experience results in a varying bias depending on the underlying sample range (Petzschner and Glasauer, 2011). The participants’ behavior was best explained by an iterative Bayesian estimate derived from the current noisy measurement merged with information from short-term prior experience, which is updated on a trial by trial basis.

Sensory input is often embedded not just in the temporal context of prior experience, but occurs together with other indirect cues that provide a contextual environment helping to interpret the sensory input. These indirect or symbolic cues join together with sensory input and experience to yield a uniform percept. While there is a considerable body of research on multi-modal sensory fusion, the mechanisms of integration of symbolic cues into sensory perception are less well understood.

The present work aims to clarify the role of auxiliary contextual cues on behavior that is known to be influenced by prior experience. We extended our distance production-reproduction task (Petzschner and Glasauer, 2011) to include a symbolic cue that supplied additional, but initially uncertain information about the stimulus value. The symbolic cue values were provided as a written instruction prior to each trial and indicated whether the distance to be reproduced would be “short” or “long.” The cue values corresponded to two ranges of distances. We investigated whether (1) subjects could use such a symbolic cue that provided reliable but imprecise information about the sample distances and (2) how this abstract information influenced their estimation process. To evaluate the behavioral results in the cue condition we used two control conditions that mimicked the extreme cases of cue usage. In the first control condition, we presented participants with exactly the same distances in the same order, but without the symbolic cue. In the second control condition the “short” and “long” ranges of displacements were presented in a separate order. Thus, if subjects ignored the symbolic cue, we expected that the performance in the cue condition would resemble that of the first control condition. If subjects however separated their estimates based on the symbolic cue, the behavior should be similar to the second control condition.

**Figure 1:**
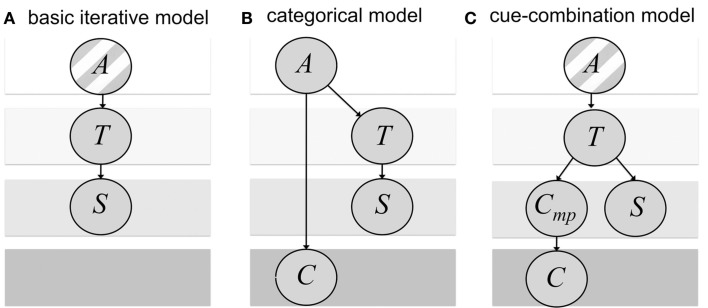
**Bayesian networks of the generative probabilistic models corresponding to the estimation part (i.e., dependence on previous trials not shown)**. The assumed probabilistic dependencies are shown as arrows. **(A)** Basic iterative model as described in Petzschner and Glasauer (2011). The stimulus *S* is a noisy measurement of the target distance *T* that is drawn from a single underlying category *A*. **(B)** Categorical model: the target distance *T* and the discrete symbolic cue *C* depend on the choice of the underlying category *A*. Again, the stimulus *S* is a noisy measurement of the target distance *T*. **(C)** Cue-combination model: The stimulus *S* and cue signal *C*_mp_ represent both independent noisy measurements of the target distance *T* that is drawn from a single underlying category *A*. The cue signal *C*_mp_ is mapped to the symbolic cue *C*. The striped background in **(A,C)** indicates that *T* is assumed to be drawn from a single category *A* in contrast to **(B)** were target distance and cue depend on the choice of the underlying category.

We then compare the behavioral data to predictions of two distinct Bayesian observer models, the *categorical* and the *cue-combination model*, which are founded on qualitatively different assumptions about the causal relationship between the sensory stimulus and the symbolic cue and consequently, about how the mapping of the symbolic cue to the stimulus dimension is learned during the experiment. Both models are based on our previously published *basic iterative model* (Petzschner and Glasauer, 2011, see Figure 1A) and generate a combined estimate of the distance to be reproduced given the observed stimulus, the symbolic cue, and prior experience. In addition, in both models Kalman filters are used to dynamically update the prior experience and to learn the relation between sensory stimulus and symbolic cue.

The two models differ in how the symbolic cue is merged with prior experience and sensory input into a distance estimate. This difference corresponds to different assumptions about the causal outside world structures between the stimulus, the measurement, and the symbolic cue (see Figure 1). In the *categorical model*, the idea is that the symbolic cue helps to identify an underlying stimulus category (Feldman et al., 2009). The model is based on the assumption that in the outside world, in each trial one of two categories is chosen, which determines the range of test distances. The test distance, which is drawn randomly from the respective category, leads to a noisy distance measurement. In addition, the symbolic cue signifies the chosen category with a certain reliability (Figure 1B). In the *cue-combination model*, it is assumed that the symbolic cue provides additional information similar to a sensory signal from a different modality (e.g., Ernst and Banks, 2002). The cue-combination model has a different view on the outside world. As our previous basic iterative model (Petzschner and Glasauer, 2011), it assumes the test distances are drawn from a single range, instead of distinct categories. The chosen test distance leads to a noisy distance measurement and to a noisy cue signal, which determines the symbolic cue (Figure 1C).

## Materials and Methods

### Participants

Twenty volunteers (nine female) aged 20–29, who had all normal or corrected-to-normal vision and were naive to the purpose of the experiments, took part in the study. Participation was monetarily compensated. The experiments were approved by the local ethics committee and conducted in accordance with Declaration of Helsinki.

### Experimental setup

**Figure 2:**
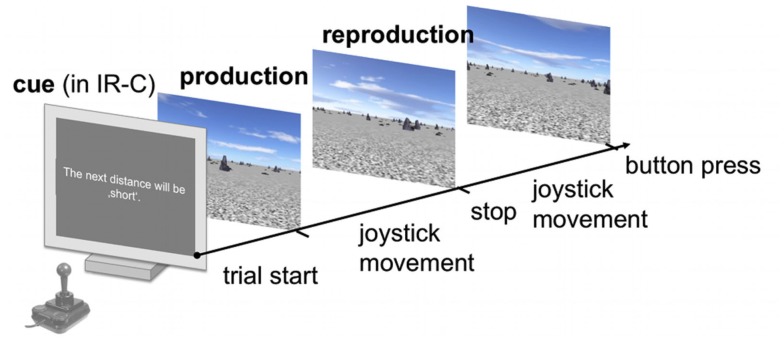
**Schematic time course of a single trial**. Subjects had to subsequently produce and reproduce a sample distance in a virtual reality using the joystick to change their position with a constant speed. The final position was indicated via button press. In the IR-C condition each production-reproduction block was preceded by a symbolic cue that declared the upcoming sample displacement to be either “short” or “long.” No symbolic cue was displayed in the IR-NC and BR-NC conditions.

Stimuli were viewed binocularly on a PnP monitor driven by an NVIDIA GeForce 8800 GTX graphics card at a frame rate of 60 Hz and with a monitor resolution of 1920 × 1200. All experiments were carried out in complete darkness except for the illumination by the monitor. The real-time virtual reality (VR) was created using Vizard 3.0 (Worldviz, http://www.worldviz.com/ and depicted the same artificial stone desert as described in Petzschner and Glasauer (2011), consisting of a textured ground plane, 200 scattered stones that served as pictorial depth cues, and a textured sky (Figure 2). The orientation of the ground plane texture, the position of the stones, and the starting position of the participant within the VR were randomized in each trial to prevent participants from using landmark cues to calibrate their estimate of displacement. The sky was simulated as a 3D dome centered on the participant’s current position and thus the distance to the horizon was kept constant. In the VR each participant’s eye height was adjusted individually to his/her true eye height. A multi-directional movable joystick (SPEEDLINK) was used to change the position with a constant speed.

### Experimental procedure

Subjects had to estimate traveled distances in a production-reproduction task in three different experimental conditions, “blocked-ranges, no cue” (BR-NC), “interleaved-ranges, no cue” (IR-NC), and “interleaved-ranges, cue” (IR-C). The task remained the same for all three conditions.

#### Task

In each trial subjects were asked to “produce” a certain sample distance, by using a joystick to move forward through the virtual environment on a linear path toward the direction of a visual object at the horizon of the virtual world until they were automatically stopped for 2.25 s. During that time they received an instruction to subsequently “reproduce” the same amount of displacement that they had experienced during the production phase. Throughout the reproduction phase subjects continued moving in the same direction as in the production phase and indicated via button press when they thought they had covered the same distance as in the production phase. In the condition with cues the symbolic cue was presented before the production phase. Figure 2 displays a schematic overview of the time course of events in a single trial. In all trials velocity was kept constant during one movement, but changed randomly up to ±60% (scaling factors between joystick output and constant VR velocity were drawn from a normal distribution) between production and reproduction phase to exclude time estimation strategies to solve the task.

#### Experimental conditions

Each experimental condition consisted of 110 trials. The first 10 trials per condition were training trials and served to familiarize participants with the task and VR. During these 10 trials, feedback on the performance was given after the reproduction phase by asking subjects to navigate toward an object that was displayed at the correct distance in the VR. The following 100 trials were test trials without any feedback. Only test trials were used for data analysis. After 50 trials subjects had a short break of 100 s to relax their hands. During that time the subjects did not leave their position and the room remained dark. Different experimental conditions were separated by a break for no less than 15 min outside the room of the experiment. In all three conditions the overall number of repetitions for each sample distance remained the same, thus the overall distribution of samples was the same for all three conditions. The same trial order within one condition as well as the same order of cues in the cued condition was maintained for all participants. The three experimental conditions were performed in a randomized order.

##### “Blocked-ranges, no cue” condition

**Figure 3:**
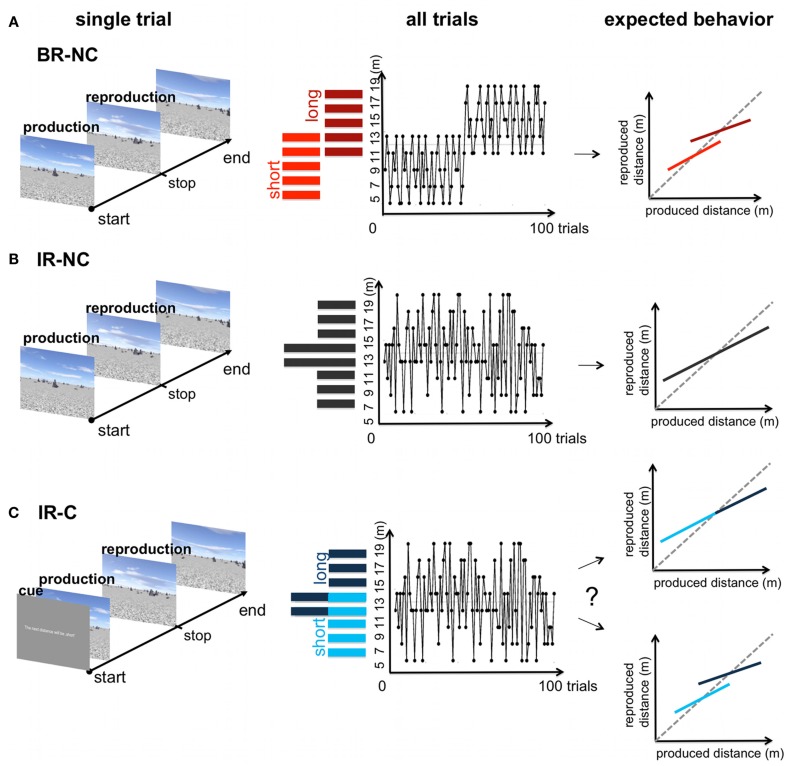
**Overview of the three experimental conditions**. Left: time course of one trial in the distance production-reproduction task. Middle: distribution and trial sequence for the blocked and interleaved-ranges. Right: Potential behavioral response. **(A)** BR-NC condition: the two sample ranges were tested in a blocked order. In the first half of the trials a range of “short” displacements was tested, in the second half of the condition a range of “long” distances was tested. Both ranges were overlapping for two distances (11 and 13 m) **(B)** IR-NC condition: The same displacements as in **(A)** where tested in the production-reproduction task, but in a interleaved order resulting in one non-uniform range of randomized sample displacements. **(C)** IR-C condition: displacements were tested in the exact same order as in **(B)**, but each trial started with a symbolic cue that indicated either a “short” or “long” displacement. No further information was provided. Depending on the influence of the symbolic cue the resulting behavior could range between the extreme cases mimicked in **(A,B)**.

In the BR-NC condition the 100 test distances were drawn in two blocks from two different underlying uniform sample distributions referred to as “short” range ([5, 7, 9, 11, 13] m) and “long” range ([11, 13, 15, 17, 19] m). In the first block of 50 trials the sample distances were randomly drawn from the “short” range distribution; sample distances for the second block of 50 trials were randomly drawn from the “long” range distribution. The two blocks were separated by a short break of 100 s. Within each range each sample distance was repeated 10 times in a randomized order. Note that the 11 and 13-m distances appeared in both the “short” and “long” range distribution, and were thus repeated 20 times in the overall condition. Thus, we refer to these displacements as overlapping samples. Subjects received no additional information about the underlying sample distribution (Figure 3A).

##### “Interleaved-ranges, no cue” condition

In the IR-NC condition the same sample distances of the two distributions were tested as in the BR-NC condition, however in a interleaved order resulting in one randomized, non-uniform sample distribution [5, 7, 9, 11, 13, 15, 17, 19] m. All samples were repeated 10 times during the overall condition, except the 11 and 13-m distance, which were again repeated 20 times. As above subjects received no additional information about the underlying sample distribution (Figure 3B).

##### “Interleaved-ranges, cue” condition

In the IR-C condition sample distances were tested in the exact same order as in the IR-NC condition based on one non-uniform sample distribution (Figure 3C). However this time subjects were told that there are two different types of samples referred to as “short” and “long” distances and that, in order to improve their performance, they would receive a written, symbolic cue that indicated which type the upcoming distance would belong to. No further information on the meaning of “short” and “long” was provided. At the beginning of each trial the sample distance was assigned on the screen to belong to one of the two types (“The next test distance will be short” or “The next test distance will be long”). All distances ranging from 5 to 9 m and one half of the 11 and 13-m distance samples were announced as being “short,” all distances ranging from 15 to 19 m and the other half of the 11 and 13-m distances were announced as being “long.” Thus the symbolic cue was always valid, except for distances 11 and 13 m, where the same distance could either be referred to as “short” or “long.” Consequently, the separation provided by the symbolic cue is comparable to the two temporally separate ranges in the BR-NC condition.

### Data analysis

Participants’ position and orientation within the VR were sampled at 20 Hz. The reproduced displacement was calculated as the difference between the position at the time of the button press and the produced displacement.

To test for differences in the behavior that are due to the use of the underlying sample range or the written symbolic cue, trials in all three conditions were split into two groups, the ranges “short” and “long.” For the BR-NC condition, where the two distributions were tested consecutively, this was achieved by splitting the trials into two halves (“short”: trials 1–50; “long”: trials 51–100). In both the IR-NC and IR-C condition trials were split according to the symbolic cue (“short” and “long”) given in the IR-C condition. Note that we also split the IR-NC condition in order to provide a direct comparison of the same trials with and without symbolic cue.

Differences in the behavioral data for the two ranges can be easily examined by comparing across those displacements that were tested in both ranges (11 and 13 m). Thus we refer to the comparison of 11 and 13 m between the “short” and “long” range as “overlapping samples comparison.”

Data analysis was conducted in MATLAB R2010b (MathWorks). Statistical differences were assessed using repeated-measures analysis of variance (rm-ANOVA). A probability level of *p* < 0.05 was considered significant for all statistical analysis. To assess differences between conditions and ranges we used rm-ANOVA for the “overlapping samples comparison” with the within-subjects factors *condition* (BR-NC, IR-NC, IR-C), *range* (“short” vs. “long”) and *distance* (two distances, 11 and 13 m). Since the use of the symbolic cue should have an effect not just on the “overlapping samples,” but also on the whole set of presented distances, we tested the difference between conditions by a second rm-ANOVA for the mean reproduction error with the within-subject factors *condition* (BR-NC, IR-NC, IR-C) and *distance* (10 distances, see “Blocked-Ranges, No Cue” Condition).

### Modeling

In our previous study we proposed a model of iterative Bayesian estimation that explained subjects performance in a distance production-reproduction task by the incorporation of prior experience into the estimation process (Petzschner and Glasauer, 2011). This basic iterative model is applied to explain the data for the two conditions without symbolic cue (BR-NC and IR-NC) in the present work (Figure 1A). For the symbolic cue condition (IR-C) the model must be extended to incorporate information that is not only driven by prior experience but the symbolic cue itself. Important for such an extension is the interpretation of the symbolic cue. Neither the symbolic cue itself nor the experimental instruction specified (1) the value or range of values in the stimulus dimension it corresponds to, and (2) the proportion of trials in which the symbolic cue is actually valid.

As mentioned in the Introduction, we propose two qualitatively different ideas how the symbolic cue could be interpreted, how the mapping of the symbolic cue to the stimulus dimension is learned, and how it is finally integrated into the estimation process. The first interpretation, referred to as categorical model, assumes that the symbolic cue *C* is an indicator for a category *A* that determines the distribution from which the target distance *T*, that is the distance to be reproduced, is being drawn (Figure 1B). This interpretation corresponds largely to the categorical model proposed by Feldman et al. (2009), except that in their model there is no symbolic cue provided to the observer. The second interpretation, referred to as cue-combination model, assumes that the target *T* is drawn from one single distribution and the symbolic cue *C* provides additional evidence about *T* just like a sensory cue from another modality (Figure 1C). Thus, this second interpretation leads to a multi-modal fusion model in which one sensory input *S*, the stimulus measurement, is continuous and the other sensory input *C*, the symbolic cue, is discrete.

In the following, the two models are described in detail. Each model has three free parameters, which are explained in the respective section. We first describe the estimation part that fuses sensory measurement, symbolic cue, and prior experience. We then separately describe the update part that implements a discrete Kalman Filter as iterative Bayesian algorithm to update cue-related priors (categorical model) or calibrate likelihoods (cue-combination model).

**Figure 4:**
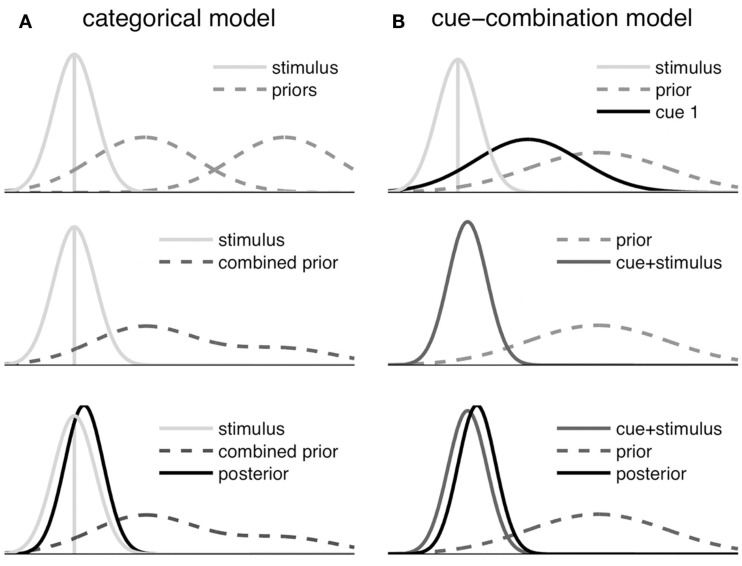
**Schematic illustration of the Bayesian fusion in the symbolic cue models**. Only the estimation step is shown, which does not include updating based on information from previous trials. **(A)** Categorical model: the category priors are merged after weighting each prior with the conditional probability of the respective category given the sensory input and the symbolic cue. Then the resulting Gaussian mixture distribution (combined prior) is fused with the stimulus measurement to derive the posterior. **(B)** Cue-combination model: first the stimulus likelihood and the likelihood corresponding to the current symbolic cue signal are fused. Then this fused signal (cue + stimulus) is combined with the prior, yielding the posterior.

The estimation part of the two models is also illustrated in Figure 4 by displaying how the prior information, the symbolic cue, and the sensory likelihood function are transformed into a posterior distribution, which determines the reproduced distance.

We use a mathematical notation where we refer to random variables with upper case letters (e.g., *A*,*S*,*T*,*C*), to values for discrete variables such as cue and category with indexed lower case letters (e.g., *c_i_*), and to values for continuous variables such as the sensory input with lower case letters (e.g., *s*). Furthermore, we abbreviate notations such as *P*(*T*,*A* = *a_i_* | *S*,*C*) to *P*(*T*,*a_i_* | *S*,*C*).

#### Categorical model

The categorical model follows Feldman et al. (2009) for the definition of the distributions. We assume that the target distance *T* is drawn from a normally distributed category

(1) $$T|A∼N\left(\mu_{A},\sigma_{A}^{2}\right)$$

and that categories *A* = *a_i_* have individual means $\mu_{a_{i}},$ but share the same variance $\sigma_{A}^{2}.$ Our generative model assumes that categories *a_i_* themselves are drawn uniformly from one of *n* possible categories (*n* = 2 in the present experiment, see Figure 4 top left). Due to measurement noise, *T* cannot be sensed directly, but only the noisy measurement *S* with the conditional Gaussian distribution

(2) $$S|T∼N\left(T,\sigma_{S}^{2}\right).$$

In addition to the direct stimulus measurement *S*, participants are presented with a symbolic cue value *c_j_*, which provides information about the underlying category. Nevertheless there is some uncertainty associated with the symbolic cue. Accordingly the cue reliability, that is, the probability of the correct symbolic cue value being presented, given a certain category *a_j_*, is specified as *p*_C_ = *P*(*c_j_* | *a_j_*) and assumed to be constant over trials. Accordingly the probability of being presented with a wrong symbolic cue out of *n−*1 remaining cues, is

(3) $$P\left(c_{j}|a_{i}\right)=\frac{1-p_{\text{C}}}{n-1}.$$

To reproduce the target distance *T*, we are interested in the posterior distribution *P*(*T* | *S*,*C*). To infer this posterior distribution, we first calculate the probability *P*(*T*,*A* | *S*,*C*), which can be derived by applying Bayes’ law to the complete joint distribution *P*(*T*,*A*,*S*,*C*), and then marginalize over the category *A*:

(4) $$P\left(T|S,C\right)=\overset{n}{\underset{i}{\sum }}P\left(T,a_{i}|S,C\right).$$

We show in the Appendix that, with the conditional dependency assumptions for this model (see Figure 1B), we can rewrite the posterior as

(5) $$P\left(T|S,C\right)=\overset{n}{\underset{i}{\sum }}P\left(T|S,a_{i}\right)\cdot P\left(a_{i}|S,C\right).$$

The category-dependent posteriors *P*(*T* | *S*,*a_i_*), which now are independent of the symbolic cue *C*, are weighted by the posterior probabilities *P*(*a_i_* | *S*,*C*) of the categories given stimulus *S* and symbolic cue *C*.

To infer the target distance we compute the mean of the posterior *P*(*T* | *S*,*C*). Analogous to the equation above, the mean of the posterior can be computed as weighted sum of conditional expectations of the category-dependent posteriors, where the weights are again the posteriors of the categories.

(6) $$E\left[T|s,c_{j}\right]=\overset{n}{\underset{i}{\sum }}P\left(a_{i}|s,c_{j}\right)E\left[T|s,a_{i}\right].$$

We show in the Appendix that this can be reformulated as

(7) $$E\left[T|s,c_{j}\right]=w_{\text{m}}s+\left(1-w_{\text{m}}\right)\overset{n}{\underset{i}{\sum }}P\left(a_{i}|s,c_{j}\right)\cdot \;\mu_{a_{i}}.$$

That is, a weighted sum of the category means $\mu_{a_{i}}$ forms the mean of a Gaussian mixture distribution (see Figure 4A middle), and this mean is summed with measurement *s* weighted by *w*_m_. The measurement weight *w*_m_ is determined by the measurement and category variances:

(8) $$w_{\text{m}}=\frac{\sigma_{A}^{2}}{\sigma_{A}^{2}+\sigma_{S}^{2}}\;\;1-w_{\text{m}}=\frac{\sigma_{S}^{2}}{\sigma_{A}^{2}+\sigma_{S}^{2}}.$$

Thus, *w*_m_ is solely determined by the ratio $\sigma_{A}^{2}∕\sigma_{S}^{2}$, which is one of the free parameters of the model. In the Appendix we show that the posteriors of the categories can be rewritten to

(9) $$P\left(a_{i}|s,c_{j}\right)=P\left(c_{j}|a_{i}\right)\cdot \alpha_{i,j}\left(s\right)$$

and thus depend on cue reliability and a measurement-dependent factor α*_i,j_*(*s*):

(10) $$\alpha_{i,j}\left(s\right)=\frac{P\left(s|a_{i}\right)}{p_{\text{C}}P\left(s|a_{j}\right)+\frac{1-p_{\text{C}}}{n-1}\overset{n}{\underset{k\neq j}{\sum }}P\left(s|a_{k}\right)}.$$

Here we exploit the specific form of the cue reliability and assume the categories to be uniformly distributed. The marginalization over *T* results in a normal distribution *P*(*S* | *A*) with

(11) $$S|A∼N\left(\mu_{A},\sigma_{S}^{2}+\sigma_{A}^{2}\right).$$

Applying the assumption for the cue reliability to the posterior expectation, we finally have

$$\begin{array}{llll} E\left[T|s,c_{j}\right] & =w_{\text{m}}s+\left(1-w_{\text{m}}\right) & & \\ & \;\times \left(p_{\text{C}}\cdot \alpha_{j,j}\left(s\right)\cdot \mu_{a_{j}}+\frac{1-p_{\text{C}}}{n-1}\overset{n}{\underset{i\neq j}{\sum }}\alpha_{i,j}\left(s\right)\cdot \mu_{a_{i}}\right). & & \text{(12)} \end{array}$$

The term within the large brackets is composed of the mean of the correct category weighted by the cue reliability and the weighted sum of all other category means.

The effect of this weighting is to select or suppress the correct category, depending on the cue reliability parameter *p*_C_. The latter would correspond to a deliberately misleading symbolic cue. Furthermore, the influence of the symbolic cue is balanced by the probability of the measurement depending on the category, which appears in α*_i,j_*(*s*).

In Feldman et al. (2009), the symbolic cue indicating the category is not provided, which corresponds to an uninformative symbolic cue. We can reflect this in our model by setting *P*(*c_j_* | *a_i_*) = 1/*n* for any *i*,*j*. We show in the Appendix that this indeed removes the dependency of the category posterior on the symbolic cue, yielding

(13) $$E\left[T|s\right]=w_{\text{m}}s+\left(1-w_{\text{m}}\right)\overset{n}{\underset{i}{\sum }}P\left(a_{i}|s\right)\cdot \mu_{a_{i}}.$$

This corresponds to Eqs 10 and 11 in Feldman et al. (2009) for equal category variance.

The posterior of *T* is a Gaussian mixture distribution, whose mean is not necessarily equal to its mode. However, the Gaussian measurement likelihood typically dominates the posterior, because its variance is small compared to the combined variance of the prior distributions corresponding to the categories. This yields a near Gaussian posterior as illustrated in Figure 4.

#### Cue-combination model

Instead of assuming that the symbolic cue signifies a category of sensory stimuli, it can also be conceived as providing additional information about the location of the stimulus in the sensory dimension. Under this assumption, the target distance *T* is drawn from a single distribution

(14) $$T∼N\left(\mu_{T},\sigma_{T}^{2}\right)$$

with the stimulus *S* being a noisy reading of *T*

(15) $$S|T∼N\left(T,\sigma_{S}^{2}\right).$$

The intuition behind the cue-combination model is that the same mechanism of multi-modal sensory fusion (e.g., Ernst and Banks, 2002), which the brain might use to combine different sensory modalities, is used to merge sensory and symbolic information. From an observer point of view, this requires an inference mechanism that maps the symbolic cue *C* to a continuous cue signal *C*_mp_. We call this signal the mapped cue. This signal is then merged with the sensory signal *S* and prior *T* in the usual Bayesian fashion. From a generative point of view, this inference inverts the causal relationships assumed for the outside world (see Figure 1C). In particular, *C*_mp_ is discretized by a step function to yield *C*. Our update mechanism, described further below, learns to map each cue value *c_i_* to a cue signal value *c*_mp_ that falls into the corresponding range. This corresponds to learning the thresholds of the step function. This mapping is deterministic, thus the cue signal becomes a known quantity, similar to actual observations. We can therefore derive the estimation step using *C*_mp_ only, leaving out *C*.

The cue signal *C*_mp_ has a likelihood function that corresponds to the average location and dispersion associated with the symbolic cue (see Figure 4B)

(16) $$C_{\text{mp}}|T∼N\left(\mu_{\text{C}}\left(T\right),\sigma_{\text{C}}^{2}\right).$$

Note that *C*_mp_ depends on *T* in a more complex way than *S*, reflected by the non-linear mapping μ_C_(*T*) We treat the cue signal *C*_mp_ the same way as the observation *S*. The mapping of the symbolic cue to the cue signal depends on the value of *C* and is updated iteratively. This updating can be understood as learning or calibration of the symbolic cue values (see Iterative update).

The optimal estimate of the target distance *T* is provided by a sensory fusion of the stimulus, the cue signal, and the prior

(17) $$P\left(T|S,C_{\text{mp}}\right)\propto P\left(S|T\right)\cdot P\left(C_{\text{mp}}|T\right)\cdot P\left(T\right).$$

With *w*_m_ as weight for the measurement *s* and *w*_fu_ as weight for the fused signal composed of mapped cue *c*_mp_ and measurement *s*, the mean for the posterior is computed as follows:

(18) $$E\left[T|s,c_{\text{mp}}\right]=\left(1-w_{\text{fu}}\right)\cdot \mu_{T}+w_{\text{fu}}\cdot \left(\left(1-w_{\text{m}}\right)\cdot c_{\text{mp}}+w_{\text{m}}\cdot s\right).$$

The weights *w*_fu_ and *w*_m_ result from the variances of target, stimulus, and symbolic cue:

(19) $$w_{\text{fu}}=\frac{\sigma_{T}^{2}}{\sigma_{T}^{2}+\sigma_{CS}^{2}}\;\;w_{\text{m}}=\frac{\sigma_{C}^{2}}{\sigma_{S}^{2}+\sigma_{C}^{2}}.$$

The combined variance $\sigma_{\mathrm{CS}}^{2}$ of symbolic cue and stimulus is

(20) $$\sigma_{CS}^{2}=\frac{\sigma_{C}^{2}\sigma_{S}^{2}}{\sigma_{C}^{2}+\sigma_{S}^{2}}.$$

Note that in the indicies we wrote *C* instead of *C*_mp_ for brevity. A more detailed derivation of the expectation of the posterior is provided in the Appendix. In short, since prior, combined likelihood, and their product are Gaussians, the mean of the posterior is given by a weighted sum of prior mean and the weighted sum of mapped cue and measurement (see Figure 4 right).

#### Iterative update

Prior experience as well as cue mapping are not available at the start of the experiment but need to be acquired and updated over the course of the trials. Such updating on a trial by trial basis can be achieved by a discrete Kalman filter updating internal states at each time step. In our case, the states correspond to the means of the two categories in case of the categorical model, to the means of the two symbolic cue likelihoods for the cue-combination model, and to the distance prior in case of the previously published basic iterative model (see Petzschner and Glasauer, 2011).

In both models, the symbolic cue is used to decide which category mean will be updated or which symbolic cue likelihood will be learned. The updating of the category means is an extension of our *basic iterative model* from one single category to multiple categories (see also Feldman et al., 2009). The iterative updating of the mean of the symbolic cue likelihood can be interpreted as learning the non-linear mapping of the symbolic cue to the stimulus dimension or as calibration of the symbolic cue in terms of a distance.

For Gaussian noise and linear dynamics, the Kalman filter yields an estimate of the current state. The current state is estimated based on the current observation and the estimate of the state at the previous time step, taking into account a deterministic temporal evolution of the state. The state *x* to be updated and the current measurement *y* at trial *i* are described by the system equations

(21) $$\begin{array}{c} x_{i}=x_{i-1}+n_{q}\\ y_{i}=x_{i}+n_{r}. \end{array}$$

The random variables *n_q_* and *n_r_* represent the process and measurement noise, which are assumed to be independent with Gaussian probability distributions *P*(*n_q_*) ≈ *N*(0, *q*) and *P*(*n_r_*) ≈ *N*(0, *r*). The temporal evolution of the state *x* defined by these equations can be seen as a random walk governed by the process noise. The measurement *y* is a noisy version of *x*.

For such a simple system, it can be shown that the difference equation system of the Kalman filter reduces to

(22) $$\begin{array}{c} k_{i}=\frac{p_{i-1}+q}{p_{i-1}+q+r}\\ p_{i}=k_{i}\cdot r\\ {\hat{x}}_{i}=\left(1-k_{i}\right)\cdot {\hat{x}}_{i-1}+k_{i}\cdot y_{i} \end{array}$$

with *k_i_* being the Kalman gain, ${\hat{x}}_{i-1}and{\hat{x}}_{i}$ being the *a priori* and *a posteriori* estimate of the state (e.g., a category mean) at trial *i*, and *p*_*i*−1_ the corresponding variance of that quantity. Note that it is evident from this equation that the Kalman gain *k_i_* can be interpreted as weight of the measurement depending on measurement noise and the assumed random change of the estimated quantity, such as a category mean. The new estimate is thus a weighted sum of the previous estimate and the current measurement.

The update for the categorical model employs a Kalman filter for each category mean to be estimated, yielding equations indexed by *j*:

(23) $$\mu_{a_{j},i}=\left(1-k_{i}^{j}\right)\cdot \mu_{a_{j},i-1}+k_{i}^{j}\cdot s_{i}.$$

For two categories we consequently have two Kalman filters, one for each category mean. The variances $\sigma_{A}^{2}$ and $\sigma_{S}^{2}$ correspond to quantities *p_i_* and *r*, respectively. Note that the ratio of the two variances only depends on the ratio *q*/*r*, which is one of the free model parameters.

The cue-combination model uses three Kalman filters to calibrate the two symbolic cue likelihoods and to update the prior for the target distance *T* using the same general form of update equations as described above.

$$\begin{array}{llll} c_{\text{mp},i}^{j} & =\left(1-k_{i}^{j}\right)\cdot c_{\text{mp},i-1}^{j}+k_{i}^{j}\cdot s_{i} & & \text{(24)}\\ \mu_{T,i} & =\left(1-k_{i}^{T}\right)\cdot \mu_{T,i-1}+k_{i}^{T}\cdot s_{i}. & & \text{(25)} \end{array}$$

The calibration of the symbolic cue likelihoods yields the mapped cues used in the estimation.

#### Logarithmic stimulus representation

There is some indication that magnitudes are internally represented in the brain on a log-scale (Fechner, 1860; Dehaene, 2003; Jürgens and Becker, 2006; Stocker and Simoncelli, 2006; Durgin et al., 2009). In Petzschner and Glasauer (2011) we showed that defining a Bayes-optimal observer on log-scales leads to an elegant combination of Steven’s power law with the Weber–Fechner law (Fechner, 1860; Stevens, 1961). The estimates in our models in the present work are again computed based on simplified logarithmic representations of the presented stimuli. In conjunction with that stands an additional parameter that can represent different optimal decision strategies in subjects. We shortly recap the idea here and refer to Petzschner and Glasauer (2011) for a detailed treatment. The logarithmic representation is given as

(26) $$s=ln\left(\frac{d_{\text{m}}}{d_{0}}\right)+n_{\text{m}}.$$

The internal representation of the measurement *s* is computed as the natural logarithm of the measurement on linear scales, *d*_m_. In the present work, *d*_m_ is given in virtual meters. To achieve a unit-less representation, *d*_m_ is normalized with the small constant *d*_0_ ≪ 1. The random variable *n*_m_ represents the normally distributed measurement noise $P\left(n_{m}\right)\approx N\left(0,\sigma_{S}^{2}\right).$

The estimate *x*_est_, corresponding to *E*[*T* | *s*,*c_j_*] for the categorical model and *E*[*T* | *s*,*c*_mp_] for the cue-combination model, is a log-scale value. It is transformed back to a linear scale with

(27) $$d_{r}=e^{x_{\text{est}}+\Delta x}\cdot d_{0}.$$

The result is the linear scale reproduction *d_r_* in virtual meters. We assume here that, apart from this transformation and possibly additional noise, the reproduction in subjects corresponds to the estimate.

The value Δ*x* accounts for different decision strategies of the subjects. A decision strategy collapses the posterior distribution into a single value, the estimate, which is optimal in the sense that it minimizes the expected loss due to the deviation from the real value (the real distance in our case). Typical decision strategies use the mean, median, or mode of a distribution as optimal (loss-minimal) estimate, which correspond to three typical loss functions (Körding and Wolpert, 2004). While these values are equal for normal distributions, they are different in our case, since the normal distribution transfers into a log-normal distribution after back-transformation. For the log-normal distribution mean, median, and mode differ by a linear shift of *x*_est_. Therefore, by introducing an additional parameter Δ*x* in our models, we account for different types of loss functions. We call this parameter the shift term.

#### Model fit

To analyze how well our models explain the experimental results, we fitted their free parameters such that the difference between model output and subject responses was minimized.

The free parameters in the categorical model are the cue reliability *p*_C_, the ratio $\sigma_{A}^{2}∕\sigma_{S}^{2}$ of the noise in the target distances and the measurement noise, and the shift term Δ*x* reflecting the loss function of the Bayesian estimator. The ratio $\sigma_{A}^{2}∕\sigma_{S}^{2}$ determines the weight of the measurement *w*_m_ relative to the category priors. This weighting schema reflects how subjects may put more weight on whichever quantity has less variance.

The free parameters of the cue-combination model are the shift term Δ*x* and two ratios. The first is the ratio of target distance noise to the combined noise in measurement and continuous cue signal, $\sigma_{T}^{2}∕\sigma_{S}^{2}.$ The second is the ratio of the noise in the cue signal to the measurement noise, $\sigma_{C}^{2}∕\sigma_{S}^{2}.$ Analogous to the categorical model, these ratios determine the relative weights *w*_fu_ and *w*_m_, respectively. The first is the weight of the combined measurement and cue signal relative to the prior, the second the measurement weight relative to the cue signal.

The basic iterative model has two free parameters, the shift term Δ*x* and the ratio of target distance noise to measurement noise, $\sigma_{T}^{2}∕\sigma_{S}^{2}.$ This ratio determines the measurement weight *w*_m_ of this model.

For the IR-C condition we fitted the category and cue-combination models to the responses of each single subject. That is, for each subject two sets of parameters were generated, corresponding to the two models. For the other two conditions, our models reduce to the iterative Bayesian estimation model (Petzschner and Glasauer, 2011), which we fitted in these cases. All models were fitted by minimizing the squared differences of model output and subject response in each trial using the Matlab function *lsqnonlin*.

The correct order of sample displacements over all trials in one condition was used as input to the models. Kalman filters in the models were initialized with the first observation, that is the first produced distance of the subject in the given condition.

To assess the precision of the fitted parameters, we estimated 95% confidence intervals of all parameters that were determined from the Jacobian of the parameter surface at the minimum using the Matlab function *nlparci*.

#### Model comparison

We compared the models’ goodness of fit by comparing their coefficients of determination *R*^2^. The coefficient of determination assesses the proportion of variability in the mean data that is accounted for by the respective model. To test for a significant difference in the *R*^2^ of the two model fits across subjects we used the non-parametric Wilcoxon signed rank test (Matlab procedure *signrank*).

## Results

### Behavioral data

**Figure 5:**
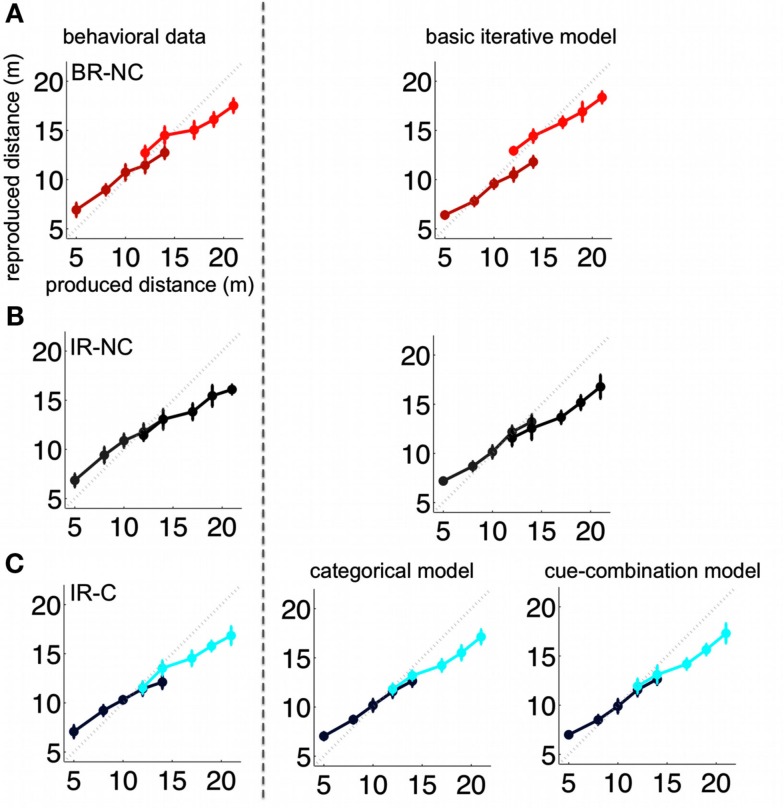
**Group mean of all subjects (left) and respective model predictions (right)**. The group mean corresponds to the mean taken over all subjects for the whole trial sequence. Models were accordingly fitted to the resulting “mean trial sequence.” The rows **(A–C)** show the results for the three conditions BR-NC, IR-NC, and IR-C respectively. The IR-NC and BR-NC were fitted with the basic iterative model introduced in Petzschner and Glasauer (2011). Predictions for the cue condition IR-C were generated with the categorical as well as cue-combination model. Error bars depict the standard deviation of the reproduced distances across trials.

In order to test the effect of an additional symbolic cue on the estimation of distances we used three experimental conditions. One condition tested the cue influence directly (IR-C condition) while the other two served as reference conditions for the extreme cases of the cue effect, i.e., ignoring the cue (IR-NC) or using the symbolic cue as perfectly reliable indicator for the stimulus range (BR-NC). The average results of all three conditions are presented in Figure 5 (left side).

Differences between conditions can be assessed by comparing the estimation of overlapping samples, that is, displacements that were assigned to the “short” as well as to the “long” distribution. However, assigning distances to a short or long range should not only affect the overlapping distances, but the estimation and consequently the reproduction errors for *all* distances presented. Condition-dependent differences in distance reproduction should occur either due to the influence of short-term prior experience or induced by the symbolic cue.

#### Comparison of distance errors

**Figure 6:**
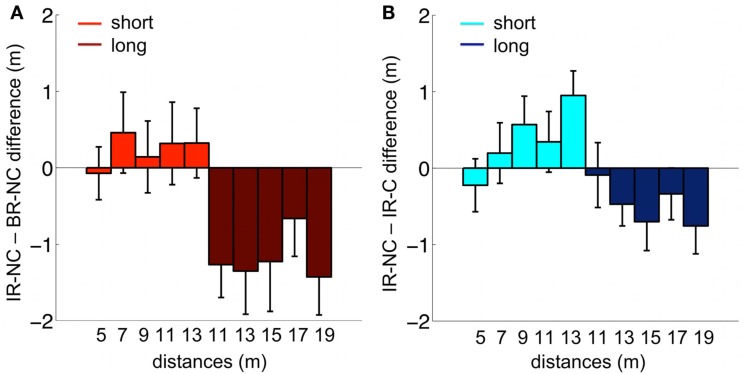
**Mean behavioral differences between conditions**. **(A)** Difference between the IR-NC and the BR-NC conditions for the mean reproduced distances. **(B)** Difference between the IR-NC and the IR-C conditions for mean reproduced distances. The only difference between the two conditions was the symbolic cue. Colors code the “short” and “long” range of displacements respectively.

The comparison of the distance reproduction error shows a main effect of *distance* [*F*(9,38) = 136.2, *p* < 0.0001] together with a highly significant interaction of *condition* and *distance* [*F*(18,342) = 3.45, *p* < 0.0001]. This interaction is due to a clear separation of error patterns between conditions, which can be seen in Figure 6 where the differences between errors in the interleaved condition (IR-NC) to the other two conditions are shown. Note that in both conditions where the ranges were separated either temporally (BR-NC) or by the symbolic cue (IR-C), the errors in the low range correspond on average to overshoots, while the errors in the high range correspond to undershoots with respect to those in the interleaved condition without cue (IR-NC). This correspondence of error patterns also confirms that the symbolic cue causes changes in distance estimation analogous to those found during temporal dissociation of the two ranges. However the effect in the IR-C condition is not as strong as in the BR-NC condition. Separate *post hoc* rm-ANOVAS with only two conditions shows that for IR-C versus BR-NC this interaction vanishes [*F*(9,171) = 1.82, *p* = 0.068 n.s.], while it remains highly significant for IR-C and IR-NC [*F*(9,171) = 3.36, *p* = 0.0008]. Thus, while in IR-C and IR-NC all distance stimuli were the same in magnitude and order, the reproduced distances are clearly different, which shows that the symbolic cue was used by the subjects in a way very similar to exploiting the temporal separation of the two ranges in the BR-NC condition.

#### Overlapping samples

The results for the whole range of distances are also supported by the overlapping samples comparison, which reveals a significant interaction of *condition* × *range* (short/long) for all experimental conditions [*F*(2,38) = 11.9, *p* = 0.0001]. This implies a significant difference in the estimation of the two overlapping distances depending on the experimental condition (see also, Figure 5).

Separate ANOVAS with only two conditions revealed the individual relationships between the conditions. Differences in subjects’ behavior based solely on temporal order were determined based on the comparison of the IR-NC and BR-NC conditions (for a detailed description, see Materials and Methods). In analogy with previous results, we find a significant interaction of *condition* × *range* in the overlapping samples comparison [*F*(1,19) = 26.5, *p* < 0.001], which confirms that temporal order affects distance reproduction. By testing for the interaction between the IR-C and IR-NC condition, we assessed exclusively cue-based differences in subjects’ behavior. Again we find a significant *condition* × *range* interaction for the overlapping samples comparison [*F*(1,19) = 8.8, *p* < 0.01]. To compare performance when the sample ranges were either separated by time or symbolic cue, we performed an rm-ANOVA for the IR-C and BR-NC condition. In this case we find no significant difference between conditions in the overlapping samples comparison [interaction: *condition* × *range*; *F*(1,19) = 3.6, *p* > 0.05 n.s]. Thus, as found above, the symbolic cue leads to a behavior that resembles the performance exhibited for presenting the stimuli in ranges separated by time as in the BR-NC condition.

The *post hoc* analysis of the individual conditions supports the results of the condition comparison. The rm-ANOVA of the overlapping samples comparison reveals a significant difference for the estimation of the overlapping samples comparison in the BR-NC condition [main effect: *range* (“short” vs. “long”) *F*(1,19) = 25.7, *p* < 0.001] but no significant difference of the overlapping samples comparison in the IR-NC condition where no separation between the ranges was provided [main effect: *range* (“short” vs. “long”) *F*(1,19) = 1.3, *p* > 0.05]. Finally, the symbolic cue in the IR-C condition caused a significant difference in behavior based on the assigned range [overlapping samples comparison: main effect: *range* (“short” vs. “long”); *F*(1,19) = 9.3, *p* < 0.01].

### Modeling

Our results show that the symbolic cue significantly affects the reproduction of the stimuli in a way that is more similar to the behavior in the BR-NC condition than to the one in the IR-C condition. This raises the question how the knowledge about the symbolic cue is incorporated into the estimation process. We compare our two models by fitting them to the responses of each single subject and also to the mean responses over all subjects computed for the overall time course of trials, which we refer to as “group mean.” Figure 5 depicts this group mean and the group mean fits of our models.

#### Categorical model for condition IR-C

The categorical model assumes that the target distances presented in each trial stem from one of two categories, and that the symbolic cue informs about the given category in that trial. The three free parameters of this model, the cue reliability, the measurement weight, and the shift term, were estimated by a least squared fit (group mean fit, *R*^2^ = 0.92: *p*_C_ = 0.74, CI_95%_ = [0.70 0.78]; *w*_m_ = 0.33; *Δx* = −0.04, CI_95%_ = [−0.05 −0.03]; individual participants fit: $\bar{p_{\text{C}}}=0.76\pm 0.13,$ *range* = [0.57 1.00]; $\bar{w_{\text{m}}}=0.32\pm 0.11,$ *range* = [0.06 0.48]; $\bar{\Delta x}=-0.06\pm 0.19,$ *range* = [−0.67 0.26]). The shift terms were not normally distributed over all subjects (Lillifors test, *p* = 0.02). Yet they show a unimodal distribution with a peak close to the shift corresponding to choosing the median of the posterior distribution as an estimate.

#### Cue-combination model for condition IR-C

In contrast to the categorical model, the cue-combination model assumes that target distances are drawn from one underlying distribution and treats the symbolic cue as a second sensory input to the system. Its three free parameters are the measurement weight, the fusion weight, and the shift term. Analogous to the categorical model they were fit using a least squares method (group mean fit, *R*^2^ = 0.91: *w*_m_ = 0.38; *w*_fu_ = 0.55; *Δx* = −0.05, CI_95%_ = [−0.07 −0.03]; individual participants fit: $\bar{w_{\text{m}}}=0.39\pm 0.10,$ *range* = [0.18 0.50]; $\bar{w_{\text{fu}}}=0.54\pm 0.16,$ *range* = [0.25 0.81]; *Δx* = −0.07 ± 0.19, *range* = [−0.67 0.25]). As in the case of the categorical model, shift terms fitted for the cue-combination model were not normally distributed over all subjects (Lillifors test, *p* = 0.03), yet showed a unimodal distribution with a peak near the shift corresponding to the median.

#### Basic iterative model for conditions IR-NC and BR-NC

If the symbolic cue is abandoned, the two new models reduce to the basic iterative model. For comparison, we fitted this model on the two non-cue conditions IR-NC and BR-NC. The model has two free parameters, which have been fitted for each of these two conditions individually (IR-NC group mean fit: *w*_m_ = 0.33; *Δx* = −0.05, CI_95%_ = [−0.07 −0.03]; IR-NC individual participants fit: $\bar{w_{\text{m}}}=0.33\pm 0.13,$ *range* = [0.03 0.48]; $\bar{\Delta x}=-0.07\pm 0.22,$ *range* = [−0.67 0.37]; BR-NC group mean fit: *w*_m_ = 0.34; *Δx* = −0.04, CI_95%_ = [−0.06 −0.02]; BR-NC individual participants fit: $\bar{w_{\text{m}}}=0.33\pm 0.09,$ *range* = [0.14 0.49]; $\bar{\Delta x}=-0.04\pm 0.12,$ *range* = [−0.29 0.26]).

#### Model comparison

**Figure 7:**
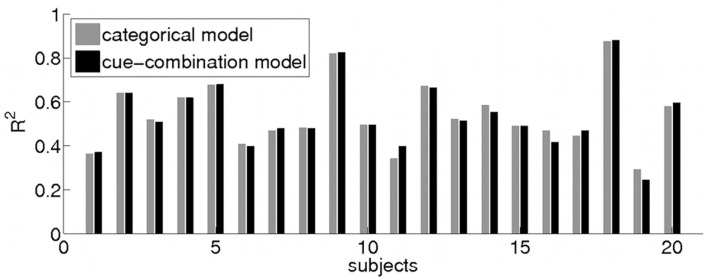
**Model Comparison**. Bar plot of individual *R*^2^ values of the model fit for the categorical (gray) and cue-combination model (black) to each subjects’ behavior (1–20) in the IR-C condition. A comparison of the goodness of fit of the categorical and the cue-combination model revealed no significant difference between the two models.

To compare the categorical and cue-combination model, we computed *R*^2^ values for individual participant fits (see Figure 7) in the IR-C condition (categorical model: $\bar{R^{2}}=0.54\pm 0.15,$ *range* = [0.31 0.88]; cue-combination model: $\bar{R^{2}}=0.54\pm 0.15,$ *range* = [0.24 0.88]) as well as for the group mean fits (categorical model: *R*^2^ = 0.92; cue-combination model: *R*^2^ = 0.91). In the other two conditions without a symbolic cue, our existing Bayesian estimator model shows similar goodness of the individual participant fits (IR-NC: $\bar{R^{2}}=0.45\pm 0.18,$ *range* = [0.05 0.72]; BR-NC: $\bar{R^{2}}=0.51\pm 0.27,$ *range* = [−0.40 0.85]). And as in the IR-C condition the group mean fit turns out to be better (IR-NC: *R*^2^ = 0.87; BR-NC: *R*^2^ = 0.88) than the individual estimates.

In comparing the goodness of fit of the categorical and the cue-combination model (non-parametric Wilcoxon signed rank test), no significant difference between the two models could be found (*p* > 0.45). We also tested whether the small differences of the subject-by-subject *R*^2^ values that can be seen in Figure 7 are related to the subjects’ response biases and variances. However, we could not find any significant correlations (Spearman ranks test, *p* > 0.13).

## Discussion

The context in which a stimulus occurs can contain additional relevant information about the stimulus itself. It is thus advantageous to combine all types of available information, in order to use the composite as an estimate of the stimulus. Here we demonstrate that this fusion of information takes place in distance estimation by path integration, where subjects incorporated prior experience and abstract information provided by a symbolic cue into their current estimate of displacement. We proposed two generative Bayesian models that describe this fusion of information based on two distinct assumptions – categorization and cue-combination.

### Cue-based range and regression effects

The influence of the symbolic cue on distance estimation behavior was assessed by comparing the cue condition (IR-C) to two reference conditions. Both mimicked the two possible extreme cases of cue usage. The no cue condition BR-NC tested two overlapping ranges of stimuli that were blocked in time, in order to change the respective prior experience of subjects and mimicked the case in which the pre-cueing by the words “short” or “long” would lead to a full separation of stimuli into two groups of events or categories. The IR-NC condition combined these two ranges to a single distribution of distances. The order and magnitude of stimuli was exactly the same as in the IR-C condition, thus replicating the cue condition for the case where the symbolic cue would be fully ignored.

In all three experimental conditions we observed a tendency to bias toward certain displacements, also referred to as regression effect (Hollingworth, 1910). In the no-cue conditions BR-NC and IR-NC the bias depended on the respective underlying sample distribution and could be explained by incorporation of short-term prior experience into the current estimate of displacements, as shown in our previous study (Petzschner and Glasauer, 2011). The behavior in the cue condition IR-C did not resemble that of the IR-NC condition although the order and size of sample displacements was the same. It was rather reflecting the behavior observed for two distinct sample ranges in the BR-NC condition, even though the effect was smaller.

Thus, the bias in the cue condition cannot be explained exclusively by the use of prior experience. This led to the question of how the additional symbolic cue information is processed. One possible explanation comes from the studies on categorization effects (Huttenlocher et al., 1991; Cheng et al., 2010). If there is uncertainty in the stimulus metric, then information about stimulus categories can be incorporated into the estimation process (Huttenlocher et al., 1991; Feldman et al., 2009). In our case, the symbolic cue could cause a sorting of stimuli into categories such that the expectation about the upcoming stimulus varies depending on whether subjects assume the stimulus to be drawn from the “short” or “long” category. We elaborated on this idea in the categorical model.

Another possible explanation, which we pursued in our cue-combination model, comes from a different field of research – multi-modal sensory cue-combination (Ernst and Banks, 2002; Ernst and Bülthoff, 2004). Similar to our findings, von Hopffgarten and Bremmer (2011) showed in a recent study on self-motion reproduction that subjects are capable of learning an abstract relationship between a novel cue and the stimulus and exploit that information to improve their performance. In their study, the frequency of a simultaneous auditory signal indicated movement speed and was used by the subjects to improve self-motion reproduction. Their study provides evidence that subjects learned the initially unknown frequency-velocity mapping provided by the auditory cue, comparable to the mapping of the symbolic cue to distance in our present experiment. Von Hopffgarten and Bremmer argued that the observed behavior could be interpreted by “sensory combination” (Ernst and Bülthoff, 2004), where the auditory input served as an additional, non-redundant cue.

### Categorical model

The categorical model is based on the assumption that the stimulus comes from one of two distinct, but perhaps overlapping, categories of stimuli, each represented by its own probability distribution (Feldman et al., 2009). Accordingly the symbolic cue provides information about the respective category. The order of events in this generative model is as follows (Figure 1B): (1) the category is chosen, (2) the information about the category is provided as symbolic cue, and (3) the stimulus is drawn from the distribution corresponding to the category. Note that the symbolic cue does not necessarily provide reliable information about the category. Hence, the prediction of the symbolic cue for a respective category is not always correct. The model represents this uncertainty with a trial-independent probability that we refer to as cue reliability.

Since the categories are unknown, they have to be learned from the symbolic cue values (“short” and “long” in the present experiment) and the stimulus presentation. Note that the semantic interpretation of the cue values is not sufficient to determine the categories, since the cue values do not specify the ranges; they only denote an order within the presented stimuli, i.e., that a “short” distance probably is shorter than a “long” one. Learning is achieved by iterative Bayesian estimation analogous to Petzschner and Glasauer (2011). Our categorical model is thus an extension of the model of Feldman et al. (2009) to explain the so-called perceptual magnet effect in speech perception. In contrast to their model, where no pre-cueing was done and the categories were assumed to be fixed, our model provides the symbolic cue values as additional uncertain information about the category and allows learning of the category means during the course of the experiment. The variance of the prior distributions could also be learned during the experiment (Berniker et al., 2010). However, in the present study we assume that it is, apart from an initialization phase, constant throughout the experiment. For other categorization tasks, such as understanding of speech, it has been proposed that the learning of weighting of acoustic cues for categorization might take place during development (Toscano and McMurray, 2010).

The combination of categorical information with the measured stimulus value was also proposed in a model by Huttenlocher et al. (1991) for estimating spatial location. In their model categorical information is used in two distinct ways. First the remembered stimulus measurement is weighted with categorical prototype information and second the resulting estimates are constrained to fall within the category boundaries. In our model estimates are not artificially restricted to certain boundaries, even though the weighting with the learned mean of the respective category will bias them toward this mean. Hence, our estimation process explains the tendency to bias toward the category means, which is reported in a variety of psychophysical studies. This *central tendency bias*, *schema*, or *range effect*, causes a tendency of estimates to be biased toward the category they where assigned to (Hollingworth, 1910; Johnson and Vickers, 1987; Cheng et al., 2010).

The category model can be extended to an arbitrary number of categories. However, introducing new categories or new cue values during the experiment would not only require learning of that category, but also re-computing of the relative weights of the other categories. In other words, a new category or new cue value should directly affect the other categories.

In the present work the number of categories is predefined and given by the number of cue values, but under many other circumstances this is not the case. Recent work (e.g., Lucas and Griffiths, 2010) addresses the question of how we determine the number of categories in the context of learning of causal structures. While this is not required in the present study, our cue-combination model, which is independent of the number of cues, may well be capable of dynamically adapting to new cue values added during the course of the experiment. This could be considered as a weaker form of structural learning.

### Cue-combination model

In contrast to the categorical model, the cue-combination model assumes that the stimulus comes from one continuous range of stimuli and the pre-cueing provides additional evidence about where in this range the current stimulus can be found. This idea is similar to common models in sensory cue-combination, where the sensory inputs from a common source are fused in order to build a unified percept of its origin (Ernst and Banks, 2002; Körding et al., 2007). In terms of a generative model, the order of events in the cue-combination model is as follows (Figure 1C): (1) the stimulus is drawn from the underlying distribution, and (2) the symbolic cue is determined from this stimulus by some mapping. In our current implementation, this mapping is assumed to be probabilistic. Therefore, a large stimulus is assumed to cause the respective symbolic cue value in most of the cases, but at some occasions it can also lead to the other cue value. Since the mapping between stimulus and cue value is not pre-specified, it has to be learned over the course of the experiment. This is achieved by iteratively adapting the mean of the likelihood function associated to each symbolic cue value. In addition to the unknown mapping, the underlying stimulus distribution is learned during the experiment (as in Petzschner and Glasauer, 2011).

A more intuitive explanation of the cue-combination model is provided from the observer point of view. Given the stimulus and an additional corresponding cue one aims to combine these two sources of information in an optimal manner. This would require that the cue can be related to a certain displacement value. This can be achieved by learning the relation between the current stimulus distance and the respective cue on a trial by trial basis. We refer to this process as mapping in the present model.

The mapping of the symbolic cue values to the stimulus dimension does not require knowledge about the possible number of cue values. Rather, the adaptation is similar to cue calibration, e.g., learning the transformation between one stimulus dimension and another (Burge et al., 2010; Zaidel et al., 2011). Thus, in contrast to the category model, adding another symbolic cue value during the experiment would not require a change in the mapping of the previously presented cues. This makes the model more flexible to changes than the categorical model.

### Model comparison

Interestingly, the results of the categorical and cue-combination model are very similar, although the underlying assumptions are substantially different. The categorical model is based on an intuitive assumption about how the stimuli presented to the subjects are generated: it assumes that there are two distinct categories, from which the stimuli are drawn. This corresponds, for example, to the categories in speech production, where a certain syllable is produced or understood based on a distinct category. The cue-combination model does not assume such an underlying structure, but rather treats the symbolic cue as additional modality. Consequently, the cue-combination model is more flexible to changes in cueing while, at least for our experiment, being equally powerful in explaining the data compared to the categorical model. The main reason for the similar performance of both models is, apart from the experimental setting, the iterative updating of the “meaning” associated with the symbolic cue, which leads to very similar sources of information regarding the range of stimuli denoted by the cues. This information is, in both models, weighted by reliability either in form of a variance associated with the symbolic cue or a probability that the symbolic cue is accurate. Thus, both models can fairly well describe the behavior observed in our experiments: our participants used the symbolic cue, they were able to associate them with the stimulus magnitude, but they did not completely trust them, as evidenced by the difference between the IR-C and BR-NC conditions.

Similarly, both models would also have performed equally well in predicting the two outcomes of cue usage mimicked in the IR-NC and BR-NC condition (Figure 3). If the information provided by the cue would not be incorporated into the estimate of the displacements this would have resulted in a cue weighting close to zero reflected by either a cue reliability that is close to 0.5 in the categorical model or a very high cue variability in the cue-combination model. An extreme cue usage, as mimicked by the BR-NC condition, would have an opposite effect on the respective parameters.

This raises the question under which circumstances the two models would make different predictions. One major difference between the two estimation processes lies in the different means of incorporating prior knowledge. Consider Figures 4A,B. While the categorical model uses a combined prior that is driven by the occurrence of all respective cues, the cue-combination incorporates a global prior that only depends on short-term prior experience of the stimuli independently of the corresponding cues. We used the parameters derived from the fit of the experimental data in this paper to test how these differences could lead to differing predictions of the cue-combination model and categorical model under specific circumstances.

Imagine the case where the two ranges are clearly separated. Due to the influence of the experience driven prior the cue-combination model would be biased by the full range of all displacements causing a global underestimation in the high range and an overestimation of the short range of stimuli. In contrary, the combined prior in the categorical model would show two discrete peaks at the center of the respective categories and thus lead to an estimate that is, for both ranges, centered closer to the single category means. However, this strong bias in the cue-combination model would only become evident if we assume a constant variance of the prior. If the variance of the prior is also updated on a trial by trial basis (Berniker et al., 2010; Verstynen and Sabes, 2011), both models would again become similar.

Yet another case in which both models differ should become obvious when omitting the cue in some catch trials. The cue-combination model would then reduce to the basic model and rely on the global unimodal prior, thus resulting in a global tendency to the mean (Petzschner and Glasauer, 2011). In contrast, the categorical model works with two prior distributions even when the cue is missing. In that case, our categorical model reduces to the category model of Feldman et al. (2009) and would exhibit the perceptual magnet effect, which biases the reproduction toward the category means.

Another difference should be observable in cases where the presentation of the cue and stimulus is not fully randomized. Consider a case where the “long” cue is repeatedly presented in a block with a long displacement. The cue-combination model would show a quick adaption of the global prior to these long displacements, which would result in reproduction values biased toward the long displacements. The categorical model would predict a much weaker adaption to the block as it still incorporates all potential cues, the long as well as the short ones. In that respect the categorical model seems to have a longer memory and less flexibility for fast changes.

Finally, both models become mathematically equivalent for a specific parameter combination. This is the case if the variance of the global prior in the cue-combination model becomes large enough and the cue reliability in the categorical model is set to unity. That is, for the categorical model we have to set *p*_C_ = 1 in Eqs 10 and 12. For the cue-combination model, we set $\sigma_{T}^{2}=\infty$ in Eq. 20 so that *w*_fu_ = 1. Then the conditional expectations for both models (Eqs 12 and 19) become equivalent.

### Iterative learning and calibration

The no cue conditions demonstrated that subjects incorporated knowledge about the stimulus history into their current estimate of displacement. We model this iterative learning of prior knowledge by a discrete Kalman filter. In our previous work we showed that this online update of prior experience explains small variation in the data that a fixed prior could not account for (Petzschner and Glasauer, 2011). That humans are indeed capable of learning not only the mean but also the variance of an experience driven prior distribution was also recently shown (Berniker et al., 2010; Verstynen and Sabes, 2011).

The significant influence of the symbolic cue on the behavioral performance in the cue condition further shows that most subjects also included this information into their estimate of displacement. As mentioned above, the semantic interpretation of the cue values was not sufficient to allow such a fusion of cue values and sensory stimulus. Thus, subjects had to learn how to associate both. The cue-combination model interprets this learning as a mapping of the cue values onto the stimulus dimension. That an abstract, even arbitrary, mapping of different types of information can be acquired during the course of an experiment was also shown by Ernst (2007). In his study subjects were trained with stimuli that usually are unrelated in the world, such as the luminance of an object and its stiffness, but which in the experiment had a fixed mapping. He showed that subjects learned to integrate the two formerly unrelated signals, similar to the mapping in our models. Calibration is, however, not only necessary between unrelated stimulus dimensions, but also between those which are normally related, such as visual and vestibular signals indicating self-motion. A recent study could show that such a calibration is independent of the reliability of the cue (Zaidel et al., 2011), which corresponds to the learning or calibration implemented in our models.

## Conclusion

Natural human action and perception profits from the incorporation of contextual information. We show that in addition to the previously found influence of prior experience, humans are also capable of using non-metric information, in the form of a symbolic cue, for their estimate of displacement, even if the mapping of the symbolic cue onto the stimulus dimension has to be acquired during the experiment. Two substantially different models of how this information enters the estimation process led to equally good fits to the experimental data. This result sheds new light on the modeling of behavioral problems such as categorization, cue-combination, and trial-to-trial dependencies.

## Conflict of Interest Statement

The authors declare that the research was conducted in the absence of any commercial or financial relationships that could be construed as a potential conflict of interest.

## Appendix

### Categorical model

Our categorical model shall infer the target distance *T* from given measurement *s* and cue *c_j_*. Here we derive the posterior distribution over *T* from known distributions, along with its conditional expectation. The posterior is given as

(A1) $$P\left(T|S,C\right)=\overset{n}{\underset{i}{\sum }}P\left(T,a_{i}|S,C\right).$$

We have to marginalize over the categories because they are unknown. The key idea is now to express the posterior as a weighted sum over distributions of which we can easily compute the expectation.

We first factorize the posterior within the sum to obtain a distribution of which we can easily compute its expectation. As it turns out, it is the posterior of *T* given *S* and the category.

(A2) $$P\left(T,a_{i}|S,C\right)=P\left(T|a_{i},S,C\right)\cdot P\left(a_{i}|S,C\right).$$

Due to model assumptions concerning the factorization, the posterior of *T* does not depend on *C* once the category *A* is given. The full joint distribution for our model, according to our assumptions (Figure 1B), factorizes as follows:

(A3) $$P\left(T,A,S,C\right)=P\left(S|T\right)P\left(T|A\right)P\left(C|A\right)P\left(A\right).$$

We use this factorization of the full joint marginalize out *T*.

(A4) $$P\left(A,S,C\right)=\int P\left(C|A\right)P\left(S|t\right)P\left(t|A\right)P\left(A\right)dt=P\left(C|A\right)P\left(A\right)\int P\left(S,t|A\right)dt=P\left(C|A\right)P\left(A\right)P\left(S|A\right).$$

Then, by applying Bayes’ theorem we, see

(A5) $$P\left(T|a_{i},S,C\right)=\frac{P\left(T,a_{i},S,C\right)}{P\left(a_{i},S,C\right)}=\frac{P\left(S|T\right)P\left(C|a_{i}\right)P\left(T|a_{i}\right)P\left(a_{i}\right)}{P\left(C|a_{i}\right)P\left(a_{i}\right)P\left(S|a_{i}\right)}=\frac{P\left(S|T\right)P\left(T|a_{i}\right)P\left(a_{i}\right)}{P\left(S|a_{i}\right)P\left(a_{i}\right)}=\frac{P\left(S|T,a_{i}\right)P\left(T,a_{i}\right)}{P\left(S,a_{i}\right)}=P\left(T|S,a_{i}\right).$$

We, see that all factors depending on *C* cancel each other out. The posterior thus does not depend on *C*, given *A*. Following the definition of the expectation, we now have

(A6) $$E\left[T|s,c_{j}\right]=\int t\overset{n}{\underset{i}{\sum }}P\left(t,a_{i}|s,c_{j}\right)dt=\int t\overset{n}{\underset{i}{\sum }}P\left(t|s,a_{i}\right)\cdot P\left(a_{i}|s,c_{j}\right)dt$$

with *s* and *c_j_* being the known distance measurement and the known cue. We, see that the posterior of the category *P*(*a_i_* | *s*,*c_j_*) does not depend on *t*, which means we can do the following reordering. We pull *t* into the sum, exchange sum and integral and pull out the posterior of the category:

(A7) $$E\left[T|s,c_{j}\right]=\overset{n}{\underset{i}{\sum }}P\left(a_{i}|s,c_{j}\right)\int t\;P\left(t|s,a_{i}\right)dt.$$

The integral expresses the expectation of the category-dependent posterior of *T*. Thus we have

(A8) $$E\left[T|s,c_{j}\right]=\overset{n}{\underset{i}{\sum }}P\left(a_{i}|s,c_{j}\right)E\left[T|s,a_{i}\right].$$

We will now express the posterior of the category, the weights in the above sum, through known distributions.

(A9) $$P\left(A|S,C\right)=\frac{P\left(A,S,C\right)}{P\left(S,C\right)}=\frac{P\left(C|A\right)P\left(A\right)P\left(S|A\right)}{P\left(S,C\right)}=\frac{P\left(C|A\right)P\left(A\right)P\left(S|A\right)}{\underset{k}{\sum }P\left(C|a_{k}\right)P\left(S|a_{k}\right)P\left(a_{k}\right)}.$$

We reused the factorization of *P*(*A*,*S*,*C*) derived above. In the denominator we use it to marginalize out *A*. All distributions appearing in the result are known from our model assumptions, except *P*(*S* | *A*). It results from integrating over *t*:

(A10) $$P\left(S|A\right)=\int P\left(S,t|A\right)dt=\int P\left(S|t\right)P\left(t|A\right)dt.$$

Since both *P*(*S* | *t*) and *P*(*t* | *A*) are normally distributed, integrating over *t* yield the following distribution:

(A11) $$S|A∼N\left(\mu_{A},\sigma_{S}^{2}+\sigma_{A}^{2}\right).$$

Remember that we assume equal variances for all categories.

A special case of this model is if no cue is present. This corresponds to a case where all cues appear with equal probability independently of the given category, *P*(*c_j_* | *a_i_*) = *1/n*, and thus tell us nothing about the category. This leads to the posterior of *A* becoming independent of *C*:

(A12) $$P\left(A|S,C\right)=\frac{1/nP\left(A\right)P\left(S|A\right)}{\sum_{k}1/nP\left(S|a_{k}\right)P\left(a_{k}\right)}=\frac{P\left(A\right)P\left(S|A\right)}{P\left(S\right)}=P\left(A|S\right).$$

With the posterior of the category now being independent of *C*, the expectation for *T* given measurement and cue in Eq. 33 above reduces to Eq. 29 in Feldman et al. (2009). The Feldman model is thus a special case of our model.

It remains to compute the expectations of the category-dependent posteriors, *E*[*T* | *s*,*a_i_*]. These posteriors result from standard Bayesian fusion of the likelihood for *S* and the prior *P*(*T* | *A*), as we have shown above:

(A13) $$P\left(T|S,A\right)=\frac{P\left(S|T\right)P\left(T|A\right)}{P\left(S|A\right)}=\frac{P\left(S|T,A\right)P\left(T|A\right)}{P\left(S|A\right)}.$$

Since both the likelihood *P*(*S* | *T*) and the prior *P*(*T* | *A*) are normally distributed, the posterior is normally distributed with mean and variance

(A14) $$\mu_{i}=\frac{s\sigma_{A}^{2}+\mu_{a_{i}}\sigma_{S}^{2}}{\sigma_{S}^{2}+\sigma_{A}^{2}}\;\;\sigma_{i}^{2}=\frac{\sigma_{S}^{2}\sigma_{A}^{2}}{\sigma_{S}^{2}+\sigma_{A}^{2}}.$$

That allows us to write the expectation as

(A15) $$\begin{array}{c} E\left[T|s,c_{j}\right]=\overset{n}{\underset{i}{\sum }}P\left(a_{i}|s,c_{j}\right)\frac{s\sigma_{A}^{2}+\mu_{a_{i}}\sigma_{S}^{2}}{\sigma_{S}^{2}+\sigma_{A}^{2}}=\overset{n}{\underset{i}{\sum }}P\left(a_{i}|s,c_{j}\right)\left(\frac{\sigma_{A}^{2}}{\sigma_{S}^{2}+\sigma_{A}^{2}}s+\frac{\sigma_{S}^{2}}{\sigma_{S}^{2}+\sigma_{A}^{2}}\mu_{a_{i}}\right)\\ =\overset{n}{\underset{i}{\sum }}P\left(a_{i}|s,c_{j}\right)w_{\text{m}}s+\overset{n}{\underset{i}{\sum }}P\left(a_{i}|s,c_{j}\right)\left(1-w_{\text{m}}\right)\mu_{a_{i}}=w_{\text{m}}s+\left(1-w_{\text{m}}\right)\overset{n}{\underset{i}{\sum }}P\left(a_{i}|s,c_{j}\right)\mu_{a_{i}}. \end{array}$$

This can be further simplified using another two of our model assumptions. First, we assume that, *a priori*, categories are uniformly distributed, that is *P*(*a_i_*) = *1/n*. Second, we assume that the correct cue appears with some probability *P*(*c_j_* | *a_i_*) = *p*_C_, *j* = *i*, while the remaining wrong cues appear with equal probabilities *P*(*c_j_* | *a_i_*) = (1 − *p*_C_)/(*n*–1). First we rewrite the posterior of the category:

(A16) $$\begin{array}{l} P\left(a_{i}|s,c_{j}\right)=\frac{P\left(c_{j}|a_{i}\right)\cdot P\left(a_{i}\right)P\left(s|a_{i}\right)}{\sum_{k}P\left(c_{j}|a_{k}\right)P\left(s|a_{k}\right)P\left(a_{k}\right)}\\ \;=P\left(c_{j}|a_{i}\right)\cdot \frac{1/nP\left(s|a_{i}\right)}{p_{C}P\left(s|a_{j}\right)1/n+\frac{1-p_{C}}{n-1}\sum_{k\neq j}P\left(s|a_{k}\right)1/n}\\ \;=P\left(c_{j}|a_{i}\right)\cdot \frac{P\left(s|a_{i}\right)}{p_{C}P\left(s|a_{j}\right)+\frac{1-p_{C}}{n-1}\sum_{k\neq j}P\left(s|a_{k}\right)}=P\left(c_{j}|a_{i}\right)\cdot \alpha_{i,j}\left(s\right). \end{array}$$

This results in

(A17) $$E\left[T|s,c_{j}\right]=w_{\text{m}}s+\left(1-w_{\text{m}}\right)\overset{n}{\underset{i}{\sum }}P\left(c_{j}|a_{i}\right)\alpha_{i,j}\left(s\right)\mu_{A_{i}}.$$

Then we can further rewrite by again replacing *P*(*c_j_* | *a_i_*) to get

(A18) $$\begin{array}{c} E\left[T|s,c_{j}\right]=w_{\text{m}}s+\left(1-w_{\text{m}}\right)\left(p_{\text{C}}\alpha_{j,j}\left(s\right)\mu_{a_{j}}+\overset{n}{\underset{i\neq j}{\sum }}\frac{1-p_{\text{C}}}{n-1}\alpha_{i,j}\left(s\right)\mu_{a_{i}}\right)\\ =w_{\text{m}}s+\left(1-w_{\text{m}}\right)\left(p_{\text{C}}\alpha_{j,j}\left(s\right)\mu_{a_{j}}+\frac{1-p_{\text{C}}}{n-1}\overset{n}{\underset{i\neq j}{\sum }}\alpha_{i,j}\left(s\right)\mu_{a_{i}}\right). \end{array}$$

### Cue-combination model

Our assumptions about the (conditional) distributions of target distance *T*, stimulus *S* and mapped cue *C*_mp_ (the cue signal) lead to the following factorization of the model’s full joint probability:

(A19) $$P\left(T,S,C_{\text{mp}}\right)=P\left(S|T\right)P\left(C_{\text{mp}}|T\right)P\left(T\right).$$

From this, the posterior follows immediately:

(A20) $$P\left(T|S,C_{\text{mp}}\right)=\frac{P\left(T,S,C_{\text{mp}}\right)}{P\left(S,C_{\text{mp}}\right)}=\alpha P\left(S|T\right)P\left(C_{\text{mp}}|T\right)P\left(T\right)\propto P\left(S|T\right)P\left(C_{\text{mp}}|T\right)P\left(T\right).$$

We can, see that the posterior density function is, apart from the proportionality factor α = 1/*P*(*S*,*C*_mp_), a product of Gaussians. First, we combine the two likelihood density functions. Following the product rule for Gaussians, this product yields a Gaussian with the following parameters:

(A21) $$\mu_{CS}=\frac{s\sigma_{C}^{2}+c\sigma_{S}^{2}}{\sigma_{S}^{2}+\sigma_{C}^{2}}\;\;\sigma_{CS}^{2}=\frac{\sigma_{S}^{2}\sigma_{C}^{2}}{\sigma_{S}^{2}+\sigma_{C}^{2}}.$$

In the indices we write *C* instead of *C*_mp_ for brevity and better readability, e.g., μ*_CS_* instead of $\mu_{C_{\text{mp}}S}$. A problem is that the (unknown) mean of the symbolic cue likelihood, μ_C_(*T*), depends non-linearly on *T*. However, we may assume that this dependence is approximately linear. Remember that our model receives as input a discrete cue, which steers a calibration process (implemented by a Kalman filter), whose output in each trial is then interpreted as additional measurement *c*_mp_ of the mapped cue. This output of the calibration process closely follows the stimuli from either the long or the short range, depending on the discrete cue. The ranges themselves do not change and therefore a normal distribution with fixed μ_C_(*T*) (after a short calibration period) can approximate the dispersion of the *c*_mp_ values.

The product of the combined likelihood density function with the density of the prior *P*(*T*) is again a product of Gaussians resulting in a Gaussian. The density of the posterior is thus Gaussian with parameters

(A22) $$\mu_{\text{posterior}}=\frac{\mu_{T}\sigma_{CS}^{2}+\mu_{CS}\sigma_{T}^{2}}{\sigma_{CS}^{2}+\sigma_{T}^{2}}\;\;\sigma_{\text{posterior}}^{2}=\frac{\sigma_{CS}^{2}\sigma_{T}^{2}}{\sigma_{CS}^{2}+\sigma_{T}^{2}}.$$

According to standard probability theory, the expectation of the posterior is then given as

(A23) $$\begin{array}{c} E\left[T|s,c_{\text{mp}}\right]=\mu_{\text{posterior}}=\frac{\sigma_{CS}^{2}}{\sigma_{CS}^{2}+\sigma_{T}^{2}}\mu_{T}+\frac{\sigma_{T}^{2}}{\sigma_{CS}^{2}+\sigma_{T}^{2}}\mu_{CS}=\left(1-w_{\text{fu}}\right)\mu_{T}+w_{\text{fu}}\mu_{CS}\\ =\left(1-w_{\text{fu}}\right)\mu_{T}+w_{\text{fu}}\frac{s\sigma_{C}^{2}+c_{\text{mp}}\sigma_{S}^{2}}{\sigma_{S}^{2}+\sigma_{C}^{2}}=\left(1-w_{\text{fu}}\right)\mu_{T}+w_{\text{fu}}\left(\frac{\sigma_{C}^{2}}{\sigma_{S}^{2}+\sigma_{C}^{2}}s+\frac{\sigma_{S}^{2}}{\sigma_{S}^{2}+\sigma_{C}^{2}}c_{\text{mp}}\right)\\ =\left(1-w_{\text{fu}}\right)\mu_{T}+w_{\text{fu}}\left(w_{\text{m}}s+\left(1-w_{\text{m}}\right)c_{\text{mp}}\right)=\left(1-w_{\text{fu}}\right)\mu_{T}+w_{\text{fu}}\left(\left(1-w_{\text{m}}\right)c_{\text{mp}}+w_{\text{m}}s\right) \end{array}$$

with the weights

(A24) $$\begin{array}{c} w_{\text{fu}}=\frac{\sigma_{T}^{2}}{\sigma_{CS}^{2}+\sigma_{T}^{2}}\;\;1-w_{\text{fu}}=1-\frac{\sigma_{T}^{2}}{\sigma_{CS}^{2}+\sigma_{T}^{2}}=\frac{\sigma_{CS}^{2}+\sigma_{T}^{2}-\sigma_{T}^{2}}{\sigma_{CS}^{2}+\sigma_{T}^{2}}=\frac{\sigma_{CS}^{2}}{\sigma_{CS}^{2}+\sigma_{T}^{2}}\\ w_{\text{m}}=\frac{\sigma_{C}^{2}}{\sigma_{S}^{2}+\sigma_{C}^{2}}\;\;1-w_{\text{m}}=1-\frac{\sigma_{C}^{2}}{\sigma_{S}^{2}+\sigma_{C}^{2}}=\frac{\sigma_{S}^{2}+\sigma_{C}^{2}-\sigma_{C}^{2}}{\sigma_{S}^{2}+\sigma_{C}^{2}}=\frac{\sigma_{S}^{2}}{\sigma_{S}^{2}+\sigma_{C}^{2}} \end{array}$$
